# Diphtheria

**Published:** 1888-05

**Authors:** 


**Diphtheria.**—The correspondent of the *American Practitianer* writes that, "thirty-six cases of diphtheria have recently been treated with resorcin spray, thirty-four of which are stated to have recovered. The mortality in the same epidemic, where this treatment was not followed, is stated to have been 93.28 per cent."

